# A Case of ALK-Rearranged Combined Lung Adenocarcinoma and Neuroendocrine Carcinoma with Diffuse Bone Metastasis and Partial Response to Alectinib

**DOI:** 10.3390/curroncol29020072

**Published:** 2022-02-03

**Authors:** Chloe A. Lim, Norbert Banyi, Tracy Tucker, Diana N. Ionescu, Barbara Melosky

**Affiliations:** 1 MD Undergraduate Program, Faculty of Medicine, University of British Columbia, Vancouver, BC V6T 1Z1, Canada; chloe.lim@ahs.ca (C.A.L.); norbert.banyi@bccancer.bc.ca (N.B.); 2 Internal Medicine Residency Program, University of Calgary, Calgary, AB T2N 1N4, Canada; 3 Department of Pathology, BC Cancer, Vancouver, BC V6T 1Z1, Canada; dionescu@bccancer.bc.ca; 4 Cancer Genetics and Genomics Laboratory, Department of Pathology and Laboratory Medicine, BC Cancer, Vancouver, BC V6T 1Z1, Canada; TTucker2@bccancer.bc.ca; 5 Medical Oncology, BC Cancer, Vancouver, BC V6T 1Z1, Canada

**Keywords:** neuroendocrine, adenocarcinoma, driver mutation, ALK, LCNEC

## Abstract

We report a rare case of stage IV pulmonary combined large-cell neuroendocrine carcinoma (LCNEC) and adenocarcinoma (ACA), both demonstrating anaplastic lymphoma kinase (*ALK*) rearrangement by IHC and FISH. This 61-year-old lifelong nonsmoking Asian woman presented with a cough, and after diagnosis and surgical treatment, completed four cycles of adjuvant cisplatin and etoposide chemotherapy. She subsequently developed recurrence with bony metastases of exclusively *ALK*-positive LCNEC. Alectinib was started, and the patient experienced a partial response.

## 1. Introduction

Large-cell neuroendocrine carcinoma (LCNEC) of the lung comprises 1% of all lung cancers [1]. Despite its classification as non-small-cell carcinoma via WHO classifications, its high mitotic rate and extensive necrosis are similar to those of small-cell lung carcinoma (SCLC). Cisplatin–etoposide doublet chemotherapy is considered an appropriate adjuvant regimen in this setting given its rare entity leading to scarce randomized clinical trial data [2,3].

Anaplastic lymphoma kinase (*ALK*) gene rearrangement is a driver mutation in lung adenocarcinoma (ACA) [4]. Few case reports describing *ALK*-positive LCNEC exist [5,6]. One described combined high-grade neuroendocrine carcinoma (NEC) and ACA with ALK 3+ immunohistochemistry (IHC) on high-grade NEC and partially on ACA with confirmation by florescence in situ hybridization (FISH). Another case described a transformation associated with acquired crizotinib resistance [7]. Previous literature demonstrated that LCNEC is a biologically heterogeneous group of tumors, often comprising distinct subsets of genotypes seen in both SCLC and NSCLC, however with no *ALK* rearrangements identified [8].

We report a rare case of stage IV pulmonary LCNEC and ACA, both harboring *ALK* rearrangement, with a response to alectinib.

## 2. Case Report

**Figure 1:**
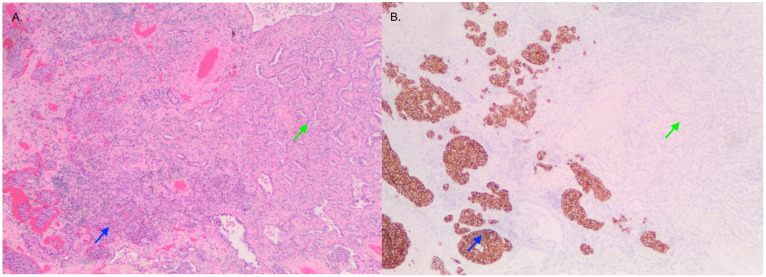
Image of tumor containing LCNEC (blue) and adenocarcinoma (green) viewed with H&E (**A**) and synaptophysin (**B**) staining that is positive in LCNEC and negative in adenocarcinoma.

A 61-year-old Asian female nonsmoker presented with a 15-year chronic cough to a pulmonologist. The patient had a prior diagnosis of primary biliary sclerosis, diagnosed in 2007, which was stable on ursodiol. Computed tomography (CT) showed 1.5 × 2.3 cm opacification within the right lower lobe (Figure 1). This was further investigated with bronchoscopy and methacholine challenge, which was inconclusive. After sequential chest CT, this nodule was found to have an interval increase in size. Therefore, the patient underwent video-assisted thoracic surgery guided with microcoil, which was converted to open right lower lobe lobectomy in July 2019 after the preliminary pathological findings. Pathology showed a 2.5 cm pT2a combined ACA and LCNEC with lymphovascular invasion. The 11R lymph node was positive for LCNEC. Final pathologic diagnosis was pT2aN1MX. Positron emission tomography scan was negative for any metastases prior to the resection.

**Figure 2:**
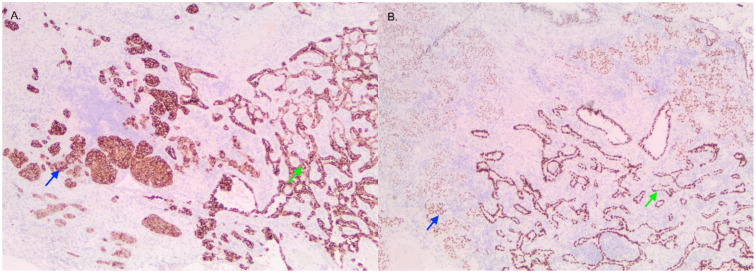
Image of tumor containing LCNEC (blue) and adenocarcinoma (green) viewed with ALK (**A**) and TTF-1 (**B**) staining. Both stains are positive in the two regions.

**Figure 3:**
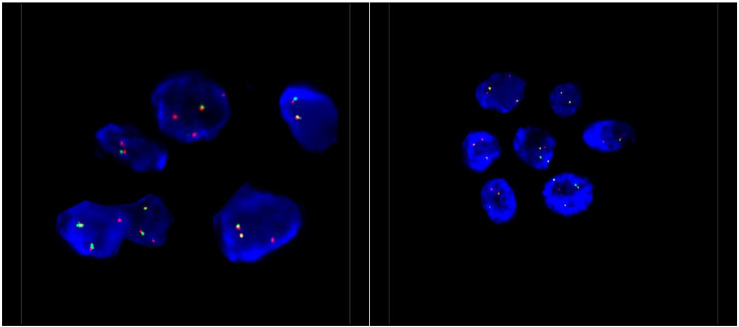
Representative *ALK* FISH images on interphase nuclei (×100 magnification). FISH analysis was performed using a dual-colour, break-apart probe (Vysis) to *ALK*, and showed 1–2 normal fused signals and one isolated 3′ *ALK* signal, which covers the kinase domain of *ALK*. This signal pattern is consistent with an unbalanced *ALK* rearrangement.

Pathological examination of the right lower lobe specimen revealed a combined LCNEC and ACA (Figure 1A). The LCNEC component had a mitotic count greater than ten mitoses per two millimeters squared. The LCNEC IHC demonstrated strong synaptophysin (Figure 1B) and CD56 cytoplasmic staining, and Ki67 staining was approximately 40%. Both LCNEC and ACA components were TTF-1 positive (Figure 2A) and negative for GATA3. ALK IHC (clone 5A4, Novocastra) (Figure 2B) showed strong cytoplasmic staining of tumor cells in both LCNEC and ACA components. FISH studies using a dual-colour, break-apart probe (Vysis^CE^) were performed, and ACA and neuroendocrine morphologies were scored separately. Signal patterns of both were similar, and consistent with an *ALK* rearrangement (Figure 3). The percentages of nuclei with the rearrangement were 65% in ACA and 48% in LCNEC.

The patient received four cycles of adjuvant cisplatin and etoposide. A CT contrast scan of the T and L spine three months later showed bony metastasis. Sclerotic lesions at T12, L1 and L5, and right iliac wing were confirmed with a bone scan. A bone biopsy of the L1 vertebral lesion was performed and confirmed metastatic *ALK*-positive LCNEC, supported by immunoreactivity for pankeratin, synaptophysin, CD56, TTF-1, and ALK. An ACA component was not present in this limited bone biopsy.

The patient was started on alectinib, an ALK inhibitor, because of disease progression despite chemotherapy. Alectinib was halted after a six-month trial due to transient transaminitis in context of her primary biliary sclerosis. With resolution of her transaminitis, she tolerated another trial of alectinib with no further increase in liver function tests. Her bone metastases have shown partial response as per RECIST radiographic criteria [9] on a dose of alectinib of 450 mg twice daily alternated with 300 mg twice daily.

## 3. Discussion

LCNEC is a rare, high-grade neuroendocrine cancer. A phase 2 study assessed optimal treatment, and demonstrated nonsuperiority of adjuvant irinotecan–cisplatin versus etoposide–cisplatin in completely resected stage I–IIIa LCNEC. Etoposide–cisplatin remains the standard first-line therapy in Canada [10]. The outcome for chemotherapy in stage IIIb and IV LCNEC is unfavorable, with median progression-free survival of 5.2 months and overall survival of 5.2 months [11].

Although *ALK* rearrangements are almost exclusively seen in ACA [4], LCNEC could also be tested for *ALK* rearrangements. Nakamura et al. investigated aberrant ALK signaling in LCNEC and found one tumor that was *ALK*-positive on IHC, but negative by FISH, and concluded that this was due to aberrant ALK expression [12]. Since this discovery, several cases of LCNEC with *ALK* rearrangement have been identified in case reports [5,6,13]. Of note, these cases did not report a concomitant ACA. Furthermore, Shimizu et al. discovered a novel *ALK* rearrangement in LCNEC resulting from a fusion of kinesin family member 5B (*KIF5B*) exon 17 to *ALK* exon 20, with positive ALK on IHC and *ALK* rearranged by FISH [14]. Previously, Rekhtman et al. identified that LCNEC displayed an NSCLC-like or SCLC-like genomic profile, highlighting that LCNEC is a biologically heterogeneous group with distinct subsets. Of note, no sensitizing *ALK* rearrangements were noted in this LCNEC cohort [8]. These studies further emphasize the significance of molecular testing of LCNEC as it likely requires a personalized therapeutic approach. The genetic diversity of LCNEC displayed by Rekhtman et al. [8] and Shimizu et al. [14], as well as previously noted ALK-positive LCNEC, may also suggest the multiclonal tumor origin [15].

The literature also describes cases where *ALK*-positive ACAs transformed to *ALK*-positive LCNEC after crizotinib treatment [7,16]. The authors speculated that neuroendocrine transformation is a potential mechanism of acquired resistance to ALK inhibitors. Another case identified an EML4::ALK rearrangement in LCNEC with crizotinib resistance [5], suggesting that perhaps *ALK*-positive LCNEC is derived from *ALK*-positive ACA via tumor plasticity.

To our knowledge, only one other case of *ALK*-positive combined lung high-grade NEC and ACA was reported in the literature. That patient was a 69-year-old female previous smoker who showed an KLC1::ALK fusion gene, and who was treated with lobectomy. She was not treated with chemotherapy or ALK inhibitors as lobectomy treated the stage IIa tumor [17].

Our case demonstrates more advanced staging with similar pathology. Thus, our patient was treated with adjuvant cisplatin–etoposide doublet chemotherapy based on the LCNEC diagnosis. This was followed by subsequent treatment with alectinib for the newly developed bony metastasis, with an ongoing partial response. This highlights alectinib’s potential efficacy in metastatic combined neuroendocrine and ACA of the lung.

A limitation to this case, is the challenge in discerning which component of the tumor responded to alectinib, taking into consideration the heterogeneity previously identified. As the bone metastases that were confirmed as metastatic *ALK*-positive LCNEC showed a partial response with alectinib, we can speculate that the LCNEC component had responded in this case.

## 4. Conclusions

Our case study highlights a rare case of stage IV combined ACA and LCNEC, both components *ALK*-positive on IHC and by FISH. With partial response with alectinib, an ALK inhibitor, this case provides insight into the potential efficacy of ALK inhibitors in certain mixed pathologies with LCNEC. Thus, next-generation sequencing to identify targetable mutations in rare histopathologies of lung cancer should be considered. Future studies exploring the efficacy of alectinib in rare mixed pathologies of lung carcinoma would aid the oncologists’ decision-making process.

## Data Availability

No new data were created or analyzed in this study. Data sharing is not applicable to this article.
